# Altered sphingoid base profiles in type 1 compared to type 2 diabetes

**DOI:** 10.1186/1476-511X-13-161

**Published:** 2014-10-11

**Authors:** Nancy Wei, Jessica Pan, Rodica Pop-Busui, Alaa Othman, Irina Alecu, Thorsten Hornemann, Florian S Eichler

**Affiliations:** Department of Medicine, Massachusetts General Hospital, Harvard Medical School, 55 Fruit Street, Boston, MA 02114 USA; MGH Neuroscience Center, Department of Neurology, Harvard Medical School, 55 Fruit Street ACC 708, Boston, MA 02114 USA; Department of Internal Medicine, Metabolism Endocrinology and Diabetes, University of Michigan Health System, 1500 E. Medical Center Drive, Ann Arbor, MI 48109 USA; Institute for Clinical Chemistry, University Hospital Zurich, Rämistrasse 100, Zürich, 8091 Switzerland; Department of Neurology, Massachusetts General Hospital, 55 Fruit Street, ACC 708, Boston, MA 02114 USA

**Keywords:** Atypical sphingolipids, Biomarker, Deoxysphingolipids, Metabolic syndrome, Type 2 diabetes mellitus, Type 1 diabetes mellitus

## Abstract

**Background:**

Sphingolipids are increasingly recognized to play a role in insulin resistance and diabetes. Recently we reported significant elevations of 1-deoxysphingolipids (1-deoxySL) - an atypical class of sphingolipids in patients with metabolic syndrome (MetS) and diabetes type 2 (T2DM). It is unknown whether 1-deoxySL in patients with diabetes type 1 (T1DM) are similarly elevated.

**Findings:**

We analyzed the long chain base profile by LC-MS after hydrolyzing the N-acyl and O-linked headgroups in plasma from individuals with T1DM (N = 27), T2DM (N = 30) and healthy controls (N = 23). 1-deoxySLs were significantly higher in the groups with T2DM but not different between T1DM and controls. In contrast to patients with T2DM, 1-deoxSL levels are not elevated in T1DM.

**Conclusions:**

Our study indicates that the 1-deoxySL formation is not per-se caused by hyperglycemia but rather specifically associated with metabolic changes in T2DM, such as elevated triglyceride levels.

**Electronic supplementary material:**

The online version of this article (doi:10.1186/1476-511X-13-161) contains supplementary material, which is available to authorized users.

## Introduction

Sphingolipids are bioactive lipids that contribute to plasma membrane and plasma lipoprotein formation. There is increasing evidence that sphingolipids play a role in insulin resistance and diabetes [1, 2]. They are derived from the aliphatic amino alcohol sphingosine, which is typically a C18 long chain (sphingoid) base and formed from the precursors L-serine and palmitoyl-CoA. This first and rate liming step in the de-novo synthesis of sphingolipids is catalyzed by the enzyme serine-palmitoyltransferase (SPT).

Besides the canonical substrates L-serine and palmitoyl-CoA, SPT is able to metabolize other acyl-CoAs in the range of C12 to C18 but also alanine and glycine under certain conditions, which forms a broad spectrum of atypical sphingoid bases. Approximately 15% of the sphingolipids in human plasma contain an atypical sphingoid base backbone [2]. In particular, the conjugation of alanine and glycine forms a class of neurotoxic 1-deoxy-sphingolipids (1-deoxSL) which lack the C1-OH group of the regular sphingoid bases. They are therefore neither metabolized to complex sphingolipids nor degraded by the canonical catabolic pathway.

Several missense mutations in SPT are associated with pathologically increased 1-deoxySL levels which causes the rare inherited sensory neuropathy HSAN1 (hereditary sensory and autonomic neuropathy type 1) [3, 4]. HSAN1 is a length dependent axonopathy, predominantly affecting the distal extremities and characterized by progressive sensory loss giving rise to painless skin ulcers and bone infections [5]. Physiologically, the de-novo sphingolipid synthesis represents a metabolic cross point which interconnects fatty acid, amino acid and thereby indirectly also carbohydrate metabolism. The observation that SPT is able to metabolize a variety of acyl-CoA and AA substrates indicates that alterations in these pathways might also be reflected by changes in the SPT product pattern.

We previously showed that 1-deoxySL levels are significantly elevated in plasma of patients with metabolic syndrome (MetS) and type 2 diabetes (T2DM) [6–8]. Here we want to extend this work by comparing the sphingoid base profile of individuals with type 1 diabetes (T1DM), T2DM and healthy controls.

## Methods

### Study participants

Blood samples were obtained through sample discards from the hospital HbA1c laboratory. We limited the sample collection to patients being seen in the MGH Diabetes Center with blood draw ICD9 codes of 250.xx, and non-Diabetes Center patient samples where HbA1c blood sample was obtained for screening purposes (V700) and where the HbA1c value was <5.4%. Chart review was performed on all patients on whom discarded samples were obtained to assess basic demographic details (age, gender), type of diabetes, duration of diabetes, co-morbid conditions including hypertension, dyslipidemia, diabetic neuropathy, insulin and statin medication use, recent blood pressure, laboratory test results (total cholesterol, triglycerides, LDL, HDL, ALT, AST, serum creatinine), and use of tobacco and alcohol. Operational definitions are provided in (Additional file 1). A chart review form was used to standardize data collection, and the data was blinded immediately after collection. Age-matched healthy controls were defined by normal glucose tolerance, normal blood pressure, normal lipids variable, and normal waist circumference. The protocol was approved by the Partners Healthcare’ Institutional Review Board, and all participants provided written consent.

### Quantification of sphingoid bases

Discarded venous blood samples were obtained in EDTA tubes and stored at 4°C until all clinical laboratory testing was complete. Plasma was obtained after spinning the samples at 3400 rpm for 15 minutes. Samples were aliquoted, anonymized and immediately frozen to -80°C. For sphingoid base analysis the samples were shipped on dry ice overnight to the University of Zurich. The sphingoid base profile was analyzed by Liquid Chromatography/Mass Spectrometry (LC-MS) after hydrolyzing the N-acyl and O-linked headgroups as described earlier [9]. Analyzed sphingoid bases included C16SO, C16SA, C17SO, C17SA, C18SO, C18SA, C20SO, C18-SA-diene, 1-deoxysphinganine, and 1-deoxysphingosine.

### Statistics

The clinical and lipid data were merged and clinical baseline characteristics and sphingoid base levels compared between groups (controls, T1DM, T2DM) by univariate and multivariate linear regression models to control for baseline differences between groups. As some of the variables measured did not follow a normal distribution, even after log-transformations, non-parametric tests were used. The Kruskal-Wallis test was used to compare all three groups, and Mann–Whitney *U* test was used for comparisons between two groups for continuous variables and X2 test for categorical variables. For multivariate analysis, sphingoid base levels were log-transformed and a generalized linear model was used to assess for significant differences between the groups initially adjusting for age, lipid levels (triglycerides, LDL), serum creatinine, and hypertension as individual covariates and building a multivariate model of variables that remained significant in the initial models (age, triglycerides, and LDL). A Bonferroni correction to account for multiple testing in multivariate model building was applied, with a significance level of 0.05 which corresponds to a p-value of 0.004. All analyses were performed with SAS statistical software, version 9.3 (SAS Institute Inc., Cary, North Carolina). The SAS multivariate linear regression model used was a proc mixed model.

## Results

For this study, we analyzed plasma samples from a total of 80 individuals with either T1DM (N = 27), T2DM (N = 30) or without diabetes or evidence of metabolic syndrome (N = 23). Unadjusted demographic and clinical characteristics are shown in Table 1. There were significant differences between the T1DM and T2DM groups. The T1DM group was in average younger and had significantly lower TG and higher HDL cholesterol levels, lower statin use and a longer disease history. Compared to controls both groups showed significantly elevated HbA1C levels indicating reoccurring phases of hyperglycemia despite glycemic control. No significant difference in HbA1c was seen between T1DM and T2DM. Nineteen of the 30 T2DM were on insulin therapy.

**Table 1:** **Baseline characteristics and results**

	Type 1	Type 2	Control	overall p-value	T1 vs. T2 p-value	T2 vs. control p-value	T1 vs. control p-value
N	27	30	23	Kruskal-Wallis/Chi-Sq	Wilcoxon/Fisher’s	Wilcoxon/Fisher’s	Wilcoxon/Fisher’s
Age, mean (s.d.)	47 (14.6)	60 (13.6)	43 (15)	<0.001	0.001	<0.001	0.4
Male, N (%)	16 (59)	16 (53)	11 (48)	0.7	0.8	0.8	0.6
HbA1c, mean (s.d.)	7.3 (1.3)	7.8 (2)	5.2 (0.3)	<0.001	0.4	<0.001	<0.001
TG, mean (s.d.)	70 (33)	145 (84)	84 (35)	<0.001	<0.001	0.003	0.1
LDL, mean (s.d.)	87 (27)	83 (35)	116 (44)	0.006	0.8	0.008	0.006
HDL, mean (s.d.)	70 (17)	49 (22)	60 (19)	<0.001	<0.001	0.02	0.04
HTN, N (%)	14 (52)	25 (83)	3 (13)	<0.001	0.02	<0.001	0.006
SBP, mean (s.d.)	124 (15)	130 (15)	125 (17)	0.2	0.08	0.3	0.8
DBP, mean (s.d.)	72 (8)	74 (8)	76 (12)	0.4	0.4	0.7	0.2
Cr, mean (s.d.)	0.97 (0.4)	0.90 (0.2)	0.91 (0.5)	0.2	0.7	0.2	0.07
Statin, N (%)	14 (52)	23 (77)	2 (9)	<0.001	0.06	<0.001	0.002
DM duration, mean (s.d.)	25 (11)	13 (8)	0		<0.001		
C16SO, mean (s.d.)	16.9 (4.4)	18.4 (6.0)	19.9 (9.5)	0.8	0.5	0.99	0.6
C16SA, mean (s.d.)	0.26 (0.13)	0.41 (0.19)	0.45 (0.22)	<0.001	<0.001	0.6	0.001
C17SO, mean (s.d.)	7.1 (1.6)	7.4 (2.3)	8.5 (3.3)	0.5	0.8	0.4	0.3
C18SAdiene, mean (s.d.)	34.9 (6.4)	30.3 (9.3)	39.3 (11.3)	0.007	0.02	0.006	0.3
C18SO, mean (s.d.)	92.3 (11.2)	87.1 (20.4)	103.9 (25.4)	0.02	0.1	0.01	0.1
C18SA, mean (s.d.)	3.1 (1.0)	2.4 (0.6)	2.9 (1.0)	0.01	0.03	0.3	0.008
C20SO, mean (s.d.)	0.13 (0.02)	0.15 (0.05)	0.14 (0.04)	0.2	0.06	0.2	0.8
C20SA, mean (s.d.)	0.02 (0.004)	0.02 (0.01)	0.02 (0.002)	0.02	0.004	0.3	0.1
doxSA, mean (s.d.)	0.05 (0.02)	0.09 (0.06)	0.05 (0.02)	<0.001	<0.001	<0.001	0.5
doxSO, mean (s.d.)	0.12 (0.04)	0.19 (0.11)	0.12 (0.04)	0.002	0.002	0.006	0.9

The sphingoid bases which are formed in the canonical SPT reaction (C18SA, C18SO, C18SA-diene) were not different between the groups although SA-diene was by trend lower in T2DM and C18SA lower in T1DM compared to controls. For the sphingoid bases with atypical chain lengths, C16SA turned out to be significantly lower in T1DM compared to T2DM or controls.

In contrast, 1-deoxySL levels were clearly different between the 3 groups. As in prior reports, the 1-deoxySLs (1-deoxysphinganine and 1-deoxysphingosine) were significantly elevated in T2DM. No difference in the 1-deoxSL levels was seen between T1DM and controls. After controlling for age, TG and LDL, the 1-deoxysphinganine levels remained significantly elevated in T2DM compared to controls (p = 0.003) whereas differences in the 1-deoxysphingosine levels were no longer significant in the multivariate analysis.

## Discussion

1-deoxySLs are atypical and neurotoxic products which are formed by SPT due to the alternate use of L-alanine over its canonical substrate L-serine. Pathologically elevated 1-deoxySL levels are formed by mutant forms of SPT and cause the rare inherited neuropathy HSAN1. However, 1-deoxySLs are typically present at low levels in the plasma of healthy individuals and significantly elevated in individuals with MetS and T2DM. This shows that also normal (wild type) SPT is able to catalyze this reaction although the reason why increased amount of these lipids are formed under metabolic conditions is not yet understood. A possible mechanism could be an increased availability of intracellular alanine in the hyperglycemic state. L-serine is formed from 3-phosphoglycerate whereas L-alanine is converted to pyruvate through alanine aminotransferase (ALT) in a reversible transaminase reaction. Hence, the precursors for both amino acids are generated during glycolysis and thereby provide a functional link between sphingolipid and carbohydrate metabolism.

In this work we compared the 1-deoxySL levels between individuals with T1DM, T2DM and healthy controls. In agreement with our earlier results we confirmed elevated 1-deoxySL in T2DM. However, 1-deoxySL levels were not increased in T1DM. The fact that glucose and HbA1c levels were not significantly different between the T1DM and T2DM groups indicates that hyperglycemia per se is not a determinant of 1-deoxySL formation. This suggests that glucose levels do not directly contribute to 1-deoxySL formation, indicating that other mechanisms are at play. However, these findings stand in contrast to the observation that 1-deoxySLs are elevated in plasma and liver of STZ rats, a T1DM animal model [6].

Alternatively, differences in the 1-deoxySL levels may be driven by elevated triglyceride levels. In fact, T2DM but not T1DM is typically associated with elevated TG levels. The correlation between 1-deoxySL levels and triglycerides has been demonstrated earlier [6], but can also be seen in the present study where 1-deoxySLs show a significant correlation to plasma TGs (see Additional file 2: Table S1). In contrast, serine-based sphingoid bases showed a closer correlation to total and LDL cholesterol levels. A functional link between plasma TG levels and 1-deoxySL formation might also explain why 1-deoxySL are elevated in the STZ rat model which have, in contrast to human T1DM patients, typically a more atherogenic lipid profile including elevated plasma TG levels. However, it is not yet clear how elevated plasma TGs affect 1-deoxySL levels. The use of alanine over serine - and not an altered lipid substrate – is at the base of 1-deoxySL formation. Yet is it unknown how plasma TG result in an altered use of alanine by SPT. It therefore remains subject of further studies to see how these two metabolic pathways are interrelated.

## Authors’ information

F.S.E. and T.H. should be regarded as joint senior authors of this work.

## Electronic supplementary material

Additional file 2: Table S1: Pearson Correlation Coefficients. (DOC 84 KB)

Additional file 1: **Operational Definitions.** (DOC 24 KB)
