# Supplementary material for: Altered sphingoid base profiles in type 1 compared to type 2 diabetes
Source: Lipids Health Dis. 2014 Oct 11;13:161. doi: 10.1186/1476-511X-13-161 (PMC4271467; doi:10.1186/1476-511X-13-161)
Supplement: Supplementary file 1 — Additional file 2: Table S1: Pearson Correlation Coefficients. (DOC 84 KB) [file 12944_2014_1168_MOESM1_ESM.doc]

Significant correlations are marked in bold (* p < 0.005; ** p < 0.0001 ).

There is a significant correlation of plasma 1-deoxySLs (dox SO and dox SA) and plasma TGs
