# Supplementary material for: Altered sphingoid base profiles in type 1 compared to type 2 diabetes
Source: Lipids Health Dis. 2014 Oct 11;13:161. doi: 10.1186/1476-511X-13-161 (PMC4271467; doi:10.1186/1476-511X-13-161)
Supplement: Supplementary file 2 — Additional file 1: Operational Definitions. (DOC 24 KB) [file 12944_2014_1168_MOESM2_ESM.doc]

**Operational Definitions:**

*Metabolic Syndrome*

The abdominal waist circumference was not available. Therefore, subjects were classified as having metabolic syndrome if they had at least 3 of the 4 National Heart, Lung, and Blood Institute (NHLBI) and the American Heart Association (AHA) conditions without meeting the ADA diagnostic criteria for diabetes mellitus:

Fasting glucose ≥100 mg/dL (or receiving drug therapy for hyperglycemia)

Blood pressure ≥130/85 mm Hg (or receiving drug therapy for hypertension)

Triglycerides ≥150 mg/dL (or receiving drug therapy for hypertriglyceridemia)

HDL-C < 40 mg/dL in men or < 50 mg/dL in women (or receiving drug therapy for reduced HDL-C)

*Type 1 and type 2 Diabetes*

Diagnoses were based purely on the billing codes used by their endocrinologist and confirmed by review of the endocrinologists clinic notes. All HbA1c measurements were performed with an HPLC method that is DCCT-aligned and serves as one of the primary reference methods for the National Glycohemoglobin Standardization Program, with intra- and inter-assay coefficients of variation less than 2% (reference Little RR, Rohlfing CL, Sacks DB. Status of hemoglobin A1c measurement and goals for improvement: from chaos to order for improving diabetes care. Clin Chem. 2011;57(2):205-14.)
